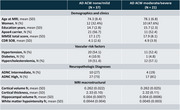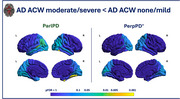# Impact of Atherosclerosis Comorbidity on Cortical Microstructure in Alzheimer's Disease Using Diffusion MRI: Evidence from an Autopsy‐Confirmed Cohort

**DOI:** 10.1002/alz70862_109733

**Published:** 2025-12-23

**Authors:** Mario Torso, Gerard R Ridgway, Pegah Khosropanah, Ian Hardingham, Steven A Chance

**Affiliations:** ^1^ Oxford Brain Diagnostics, Oxford UK

## Abstract

**Background:**

Atherosclerosis of the Circle of Willis (ACW) is a common comorbidity in Alzheimer’s disease (AD). Therefore, in the era of disease‐modifying treatments understanding how ACW may influence cortical neurodegeneration driven by AD neuropathological processes is valuable. By investigating the impact of ACW on cortical microstructural changes, we aim to identify potential cortical patterns to improve diagnostic precision, offering better‐targeted therapeutic approaches for individuals with combined vascular and neurodegenerative pathologies.

**Method:**

Structural and diffusion ante mortem MRI scans of 58 participants with intermediate or severe Alzheimer's disease neuropathologic change (ADNC) were obtained from the National Alzheimer's Coordinating Center (NACC). Participants were grouped by ACW severity (37 with none/mild and 21 moderate/severe) (Table 1).

Macrostructural MRI metrics (cortical volume and cortical thickness) and three minicolumn‐related diffusivity metrics were extracted: the angle between the radial minicolumnar direction and the principal diffusion direction (AngleR); the principal diffusion component parallel with the minicolumns (ParlPD), and the diffusion components perpendicular to the minicolumns (PerpPD^+^) (Torso et al. 2022, PMID:36281682).

The groups were compared to investigate potential differences in clinical, demographic vascular risk factors and whole‐brain MRI macrostructural data. Regional differences in macrostructural (Cortical volume and cortical thickness) and diffusion metrics were analyzed using a linear model, adjusted for the interval between the MRI scan and autopsy dates, acquisition protocol, age, and sex. The results were corrected for multiple comparisons using the false discovery rate (pFDR < 0.05).

**Result:**

Regional analysis revealed that participants with higher ACW severity exhibited a significant lower ParlPD values in the bilateral temporal and occipital regions (Figure 1), as well as significantly lower PerpPD^+^ values in the left occipital regions. No differences were found in demographic, clinical, vascular risk factor and whole‐brain MRI macrostructural data when comparing the two groups.

**Conclusion:**

These findings highlight the potential of cortical diffusivity in detecting distinct patterns of microstructural changes in individuals with the same ADNC severity but varying levels of ACW severity. This underscores the utility of cortical diffusivity for patient stratification in clinical practice and trials.